# Intercomparison on Four Irrigated Cropland Maps in Mainland China

**DOI:** 10.3390/s18041197

**Published:** 2018-04-13

**Authors:** Yizhu Liu, Wenbin Wu, Hailan Li, Muhammad Imtiaz, Zhaoliang Li, Qingbo Zhou

**Affiliations:** 1 Institute of Agriculture Resources and Regional Planning, Chinese Academy of Agriculture Sciences, Beijing 100081, China; liuyizhu1989@webmail.hzau.edu.cn (Y.L.); lizl@unistra.fr (Z.L.); zhouqingbo@caas.cn (Q.Z.); 2 Institute of Agricultural Products Processing and Nuclear Agriculture Technology Research, Hubei Academy of Agricultural Sciences, Wuhan 430070, China; hl.li@foxmail.com; 3 School of Geographical Science, Guangzhou University, Guangzhou 510006, China; m.imtiazpk92@gzhu.edu.cn

**Keywords:** irrigated cropland, GMIA, GRIPC, GlobCover, GFSAD, area comparison, spatial agreement

## Abstract

Wide-coverage spatial information on irrigated croplands is a vital foundation for food security and water resources studies at the regional level. Several global irrigated-cropland maps have been released to the public over the past decade due to the efforts of the remote sensing community. However, the consistency and discrepancy between these maps is largely unknown because of a lack of comparative studies, limiting their use and improvement. To close this knowledge gap, we compared the latest four irrigated-cropland datasets (GMIA, GRIPC, GlobCover, and GFSAD) in mainland China. First, the four maps were compared quantitatively and neutral regional- and provincial-level statistics of the relative proportions of irrigated land were obtained through regression analysis. Second, we compared the similarities and discrepancies of the datasets on spatial grids. Furthermore, the contributions of mosaic cropland pixels in GlobCover and GFSAD were also analyzed because of their extensive distribution and ambiguous content. Results showed that GMIA has the lowest dispersion and best statistical correlation followed by GRIPC, while the corresponding features of GlobCover and GFSAD are approximately equal. Spatial agreement of the four maps is higher in eastern than western China, and disagreement is contributed mostly by GlobCover and GFSAD. However, divergence exists in the ratios of the different agreement levels, as well as their sources, on a regional scale. Mosaic pixels provide more than half of the irrigated areas for GlobCover and GFSAD, and they include both correct and incorrect information. Our results indicate a need for a uniform quantitative classification system and for greater focus on heterogeneous regions. Furthermore, the results demonstrate the advantage of numerical restriction in the calculations. Therefore, special attention should be paid to integrating databases and to exploring remote sensing features and methods for spatial reconstruction and identification of untypical irrigation areas.

## 1. Introduction

Irrigated agricultural land is one of the main sources of food production. It plays an important role in water consumption and energy circulation through changing global water distribution. Therefore, it is important to understand the distribution of and changes in irrigated areas over space and time.

Owing to its wide spatial coverage and timely updating, satellite-based remote sensing has allowed continuous efforts to obtain spatial information and to assess its variation since the 1970s. In 2000, the first global irrigation map, GMIA [1,2], was released to the public. It was produced through spatial reconstruction of statistics based on the distribution of irrigation-relevant geographical features and artificial facilities. However, its accuracy varies geographically because of differences in data quality. MIRCA [3] is another map that avoids this problem through the use of uniform irrigation data. However, the method used in both GMIA and MIRCA—fusion of non-remote-sensing data—results in coarse resolution and an absence of spatial assessment. Improvements have been achieved through the use of moderate-resolution spectral images and auto-algorithms, such as GIAM/GMRCA [4,5] and GRIPC [6], which are produced by unsupervised and supervised methods, respectively. GIAM/GMRCA is a coupled map of rainfed and irrigated croplands, as well as cropping systems and water resources. The accuracy of identification of irrigated croplands ranges from 70% to 80%, depending on water resources. The uniqueness of GRIPC lies in its consideration of statistics, which results in good performance in correlation analysis with FAOSTAT, although it has lower spatial accuracy (69%) than GIAM/GMRCA. Irrigation information is also included in some datasets for plantation and land cover detection, e.g., GFSAD [7] and GlobCover [8,9], but accuracy assessments are not available for subclasses because of their complex classification systems.

These products have played an important role in scientific researches and applications. Mapping communities treat them as references against which to contrast the accuracy of novel methods, such as in the work by Zhu et al. [10], Dong et al. [11], and Salmon et al. [6]. Further, they are primary inputs in calculations of other metrics [12,13,14,15,16], e.g., estimating gross primary or crop yield, establishing land surface models, and conducting land use analysis, which has highlighted their limitations in bringing uncertainties in modeling and the final outputs of the metrics calculated using the datasets. Data quality reports such as GMIA and GRIPC reveal a possibility of regional or national superiority on accuracy of irrigation maps, which may lead to in homogenous outcomes based on them as well. In this regard, intercomparison of products could help with their improvement and selection for producers and users. However, there have been few comparison studies on irrigation maps, even though comparative procedures have been completed in similar fields such as land cover, cropland, and forests [17,18,19,20,21]. To close this gap, four typical, recent datasets were chosen for comparison in this study. The Chinese mainland was selected as the study area because of the presence of multiple forms of agriculture.

## 2. Materials and Methods

### 2.1. Four Irrigated Cropland Datasets

Summary information for the four datasets is listed in Table 1. GMIA is the only dataset obtained by a non-remote-sensing data fusion technique. Two types of irrigation areas are provided in GMIA: the area of effective irrigation (AEI) and the area of actual irrigation (AAI). We used the former because of its “good quality,” as acknowledged in the data-quality assessment report [2]. Values of each pixel represent the percentage of AEI in the pixel. The other three datasets are binary. GRIPC has a special focus on paddy fields, but this class has no description regarding irrigation status. Therefore, we treated it as irrigated land in this study because there is evidence to support the need for irrigation to provide flooding for paddy cultivation in China [22]. The classification system used by GlobCover is compatible with the GLC2000 global land cover classification [23], which is also associated with a legend defined and documented using the UN LCCS. Versions for 2005 and 2009 have been released separately; we selected the former to match the benchmark year of the other datasets (Glob05 for short). GFSAD is an integration of different trials [5,24,25,26] for the analysis of global food-supporting projects. It includes images created around 2005; therefore, we deem it comparable with GMIA, GRIPC, and Glob05, although the declared benchmark year is 2010. Both Glob05 and GFSAD have mosaic cropland pixels without irrigation information, which required additional treatment (Table 1).

### 2.2. Comparion Method

Classification systems and resolutions of different datasets need to be unified before comparison. Only irrigation-relevant classes were included in this study. Because of the absence of a rainfed class and the meaning of grid values in GMIA, the resolutions of the other maps were converted to 5 arcminute using a zonal statistical method, i.e., the value of each 5 arcminute grid is the same as GMIA, which is calculated by dividing the irrigated area by a grid’s area. As the land surface is always a mixture of different land covers, even in “pure” pixels, a simple method was used to assign the fraction of irrigated cropland [28]. We followed Xiao et al. [29] and Velpuri et al. [27] in fractional assignment of grids with different resolutions. Preprocessed Glob05 and GFSAD with separated mosaic cropland pixels (MCPs) were also taken into account for unclear definitions of MCPs with regard to irrigation (Table 1), they are labeled as “Glob05-P” and “GFSAD-P” for short in the following parts of this paper.

The comparison was conducted in quantitative and spatial phases. In phase 1, the national irrigated area was calculated by summing the area in each province. The proportion of the irrigated area replaced the absolute area on regional and provincial scales owing to double-counting caused by mosaic pixels. The country was divided into six regions and data were computed based on the provincial statistics. The statistics of the benchmark year were used as reference for neutral provincial-scale assessments. The root mean square error (RMSE) and the coefficient of correlation index (R) between the statistics from the Chinese Yearbook 2010 and the estimated data were used to reflect the dispersion and goodness-of-fit between provinces, as computed using the following equations:

(1) $$\mathrm{RMSE}=\sqrt{\sum_{i=1}^{n}{\left(x_{i}-y_{i}\right)}^{2}/n\;},$$

(2) $$R=\sum_{i=1}^{n}(x_{i}-\;\bar{x})\left(y_{i}-\;\bar{x}\right)/\sqrt{\sum_{i=1}^{n}{\left(x_{i}-\;\bar{x}\right)}^{2}\sum_{i=1}^{n}{\left(y_{i}-\;\overset{\text{\_}}{y}\right)}^{2}}$$

where $x_{i}$ and $y_{i}$ and are the proportions of irrigated area and the deviation between the dataset and statistics for province i, respectively. Correspondingly, $\bar{x}$ and $\bar{y}$ and are the means of $x_{i\;}$ and $y_{i}$, respectively, and n is the number of provinces included (n = 31). Slopes and intercepts of fitting lines were also taken down as the symbolization of their deviation level with ideal quantitative result.

Phase 2 used the same grids as phase 1. Among them, those with proportions of irrigated area >0% were treated as irrigated, which allowed each product to be converted into a binary map: 1 means irrigated and 0 means nonirrigated. An agreement map was obtained by overlaying all converted maps such that a higher pixel value (ranging from 1 to 4) represented higher agreement. Pixels with values of 1–4 were defined as having no agreement, partial agreement, high agreement, and total agreement, respectively. Analysis was also summarized both nationally and regionally.

## 3. Results

### 3.1. Visual Observation of Four Maps

**Figure 1:**
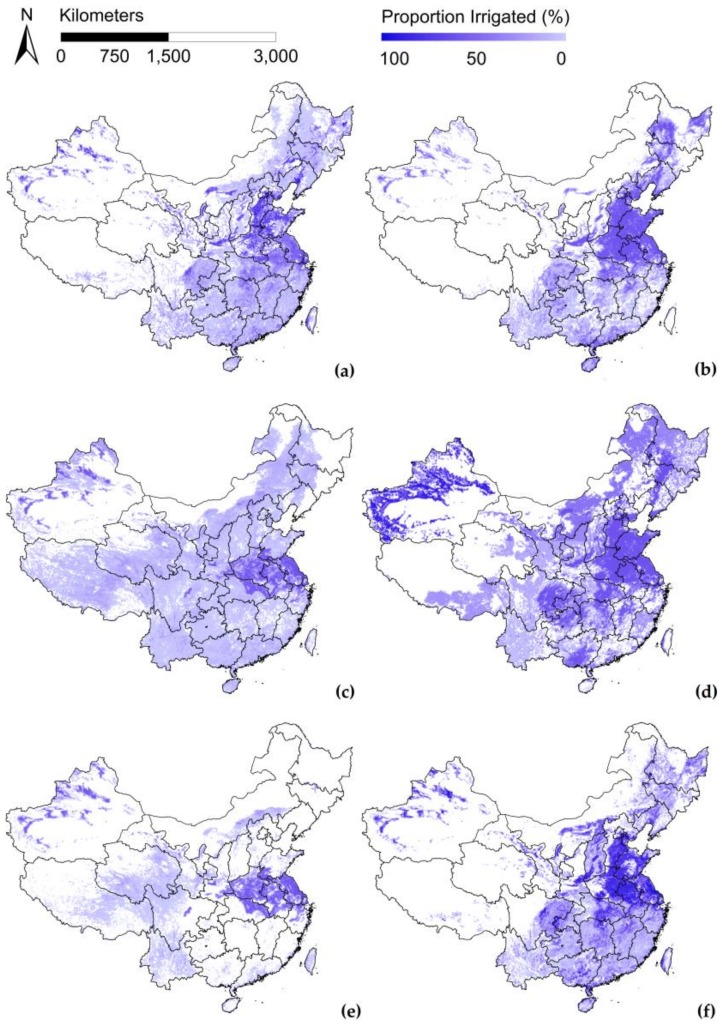
Four irrigated croplandmaps. (**a**) GMIA (**b**) GRIPC (**c**) Glob05 (**d**) GFSAD (**e**) Glob05 without mosic cropland pixels (f) GFSAD without mosaic cropland pixels.

Figure 1 shows the four irrigated cropland maps. According to Figure 1a,b, GMIA and GRIPC can be divided into two parts by a line of demarcation that starts in western Heilongjiang Province and ends in western Yunnan Province with irrigated cropland present mainly to the east of the line. The highest intensity irrigation appears on the Huanghuaihai Plain and in some local areas in northwestern China, which are typical irrigation areas. Elsewhere, the ratio of irrigation decreases gradually with increasing fragmentation of croplands. GRIPC presents a similar tendency to GMIA, while Glob05 and GFSAD have relatively high homogeneity and a wider distribution of irrigation than GMIA and GRIPC. Exclusion of MCPs alters the maps of Glob05 and GFSAD (Figure 1e,f).A dramatic reduction of the irrigated proportion of the grids is observed in Glob05, except in southern parts of the Huanghuaihai Plain and in the Xinjiang municipality. Reductions occur in GFSAD on a smaller scale; visually, typical irrigated areas appear unchanged.

### 3.2. Quantitative Comparison

#### 3.2.1. Ratios of Irrigated Croplands

**Figure 2:**
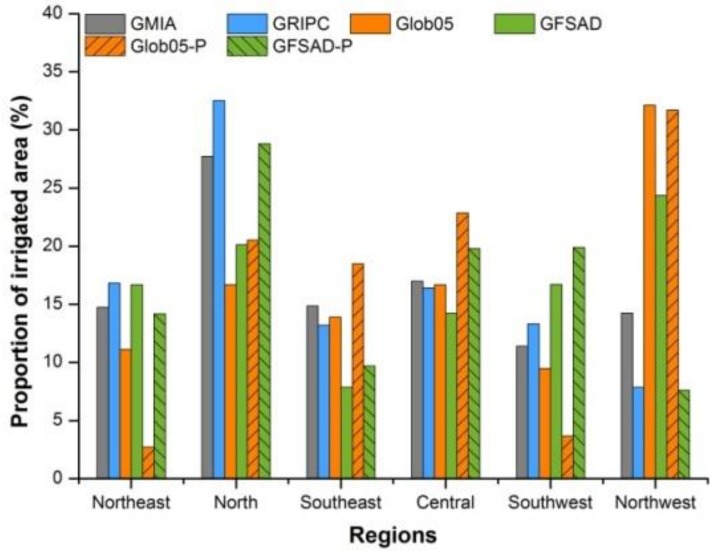
Regional ratios of irrigated croplands.

The national irrigated area in GMIA, GRIPC, Glob05, and GFSAD is 623,924, 767,212, 626,563, and 1,281,342 km^2^, respectively. The proportion of each region occupied is shown in Figure 2.

For GMIA, the northern region has the highest proportion of irrigated area (27.74%), followed in descending order by the central, northeast, southeast, northwest, and southwest areas. The greatest difference among the regions is 5.58%. The same order of regions is also presented by GRIPC but with wider variation: the north has higher and the northwest lower proportions of irrigated land than GMIA. The highest irrigation ratio for Glob05 and GFSAD appears in the northwest region (1/3 and 1/4, respectively). The central region has only slight fluctuations in regional irrigation ratio; the fluctuations are much stronger in other regions and they are mostly caused by Glob05 and GFSAD.

The values of RMSE and R, which are indicators of the dispersion and correlation of the provincial comparison, are presented in Table 2. GMIA holds the minimum of RMSE and maximum of R among the four maps, which suggests its lowest dispersion and highest goodness-of-fitting, respectively. GRIPC, GFSAD and Glob05 follow GMIA orderly for their incremental RMSE and degressive R.

**Figure 3:**
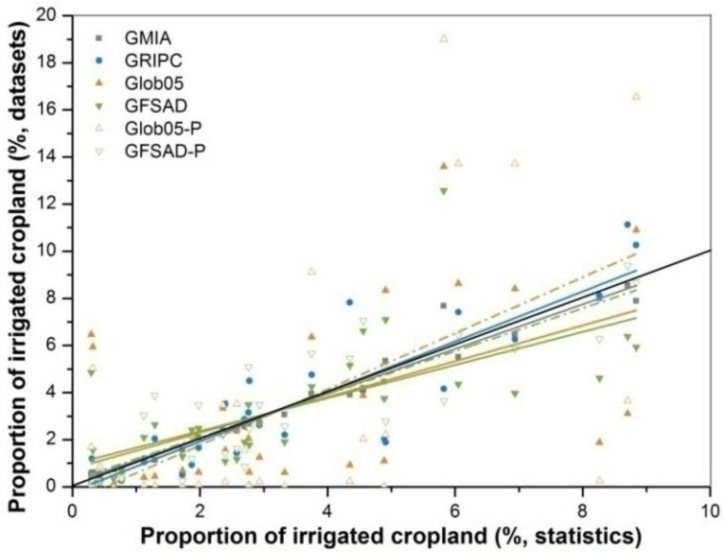
Regression of provincial proportion of irrigated cropland. Solid points correspond to solid lines of the same color, while the hollow ones correspond to dashed lines of the same color. The solid black line is the ideal line (slope = 1, intercept = 0) that symbolizes ideal dispersion and fitting.

In Figure 3, the descriptions above are presented more vividly through regression lines fitted to the raw data points. The points for GMIA are distributed mostly on or near the line of the ideal line (Slope = 1, intercept = 0), indicating that GMIA has the lowest dispersion and the best fit (which will result in accurate results for the target land use class). GRIPC is the dataset that performs next best. By comparison, Glob05 and GFSAD have the greatest dispersion and poorest fit because few points are on or near the ideal line.

Slopes and intercepts of fitting lines, which are indicators of fitting lines’ deviation with ideal line, are also displayed in Table 2 and Figure 3. Because of the internal connection of provincial proportions of AEI, the relationship between actual and ideal fitting lines can be treated as a teeterboard, the pivot of which is the intersection of the two lines. That is, a slope less than 1 and an intercept larger than 0 are always bundled together, which reveals a teeterboard leaning to data points on the left of the intersection. In other words, provinces hold those points higher in AEI proportions in product than they are in statistics in a holistic view, e.g., GMIA, GlobCover and GFSAD. Accordingly, a reverse situation is indicated by a combination with slope more than 1 and a negative intercept, displayed by the fitting line of GRIPC.

#### 3.2.2. Influence of MCPs

The national irrigated area of Glob05 decreases markedly from 626,563 to 367,918 km^2^ (decrease of 41.28%) when MCPs are excluded and the extent of the corresponding decrease of GFSAD is 32.51%. Alterations in regional areas when accounting for MCPs are given in Table 3.

Accounting for MCPs inevitably alters the ratio of regional and provincial irrigated land (Figure 2 and 3 and Table 2). The ratio increases in the north, southeast, and central regions in Glob05. For GFSAD, the ratio increases in the southwest. This leads to a fluctuation in the weight of each region for overall irrigation, especially for the northeast in Glob05 and the northwest in GFSAD. A higher dispersion and unchanged goodness-of-fit for Glob05 with MCPs excluded is indicated by a higher RMSE and a reasonably stable R value. However, both dispersion and goodness-of-fit are improved for GFSAD when MCPs are excluded, as indicated by the increased values of RMSE and R. However, the altered slopes and intercepts of fitting lines for both maps suggest that provinces with high AEI proportions present even more holistic view. Meanwhile, absolute values of the differences between slopes or intercepts with ideal ones reveal a closer distance between actual and ideal fitting lines.

### 3.3. Spatial Comparison

#### 3.3.1. Spatial Agreement

**Figure 4:**
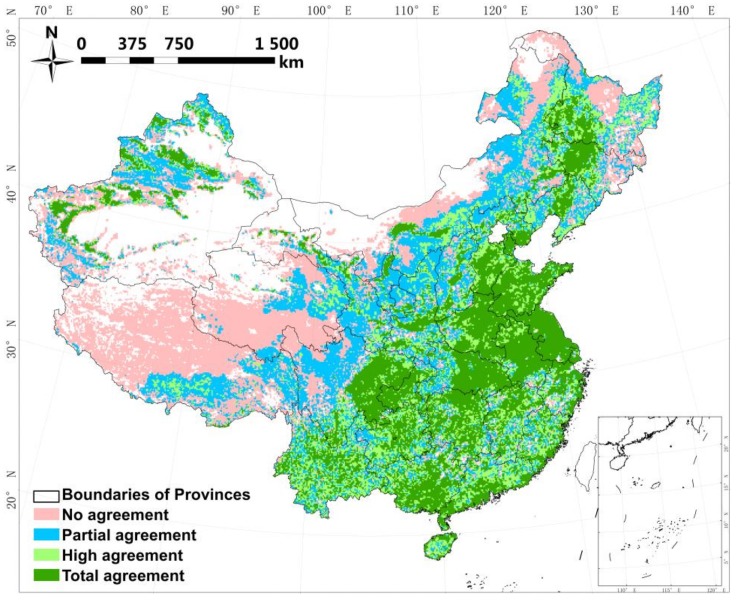
Spatial agreement of datasets. No agreement means a grid is considered as irrigated by only one dataset, whereas partial, high and total agreement means a grid is considered as irrigated by two, three, or four datasets, respectively.

Figure 4 displays the spatial agreement of all four datasets. The distribution of grids with different agreement levels is similar to previous cropland comparisons in China [30]. The appearance of grids scoring 3 or 4 is coincident with key irrigated areas such as the Sanjiang and Huanghuaihai plains, Sichuan Basin, and northwestern irrigation engineering. In other places, overall, the level of disagreement increases gradually with increasing fragmentation or distance from key irrigated areas, e.g., the transitional zone between the Huanghuaihai Plain and the northwest, as well as in southern regions.

**Figure 5:**
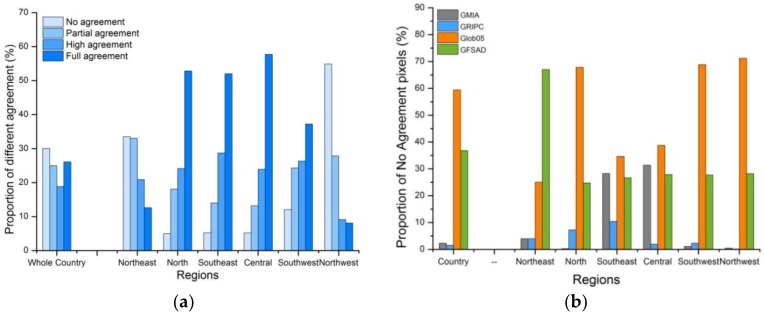
Summary of pixels with differing levels of agreement. (**a**) The proportions of pixels with different agreement levels in a holistic and regional view separately. (**b**) How much each dataset contributes regionally and nationally to disagreement.

The proportions of grids with the various levels of agreement are displayed in Figure 5a. Nationally, they range from 19% to 30% with pixels scoring 1 and 3 presenting the maximum and minimum values, respectively. Nevertheless, the components of pixels on a regional scale do not show the same pattern. The levels of agreement of the grids increase with pixel amounts in the central, north, southeast, and southwest regions, whereas they decrease in the other two regions. The highest level of agreement is found in the central region, which benefits from the greatest proportion of pixels with total agreement (score = 4). The region with the lowest level of agreement is the northwest, with >80% of grids in this region classified as partial or no agreement.

Figure 5b displays by how much each region contributes to the level of disagreement. More than 60% of pixels scored 1 are attributable to Glob05, while GFSAD contributes nearly 35%. These two datasets are the main regional contributors of disorder. In comparison, the discrepancies in GRIPC and GMIA are negligible in most regions, although the latter does not perform any better than Glob05 or GFSAD in the southeast and central regions.

#### 3.3.2. Influence of MCPs

**Figure 6:**
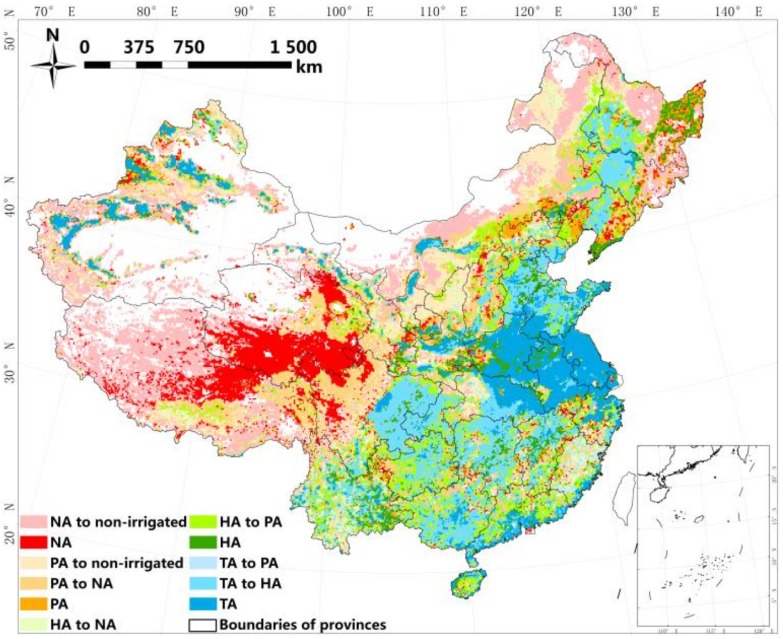
Alteration of agreement following exclusion of mosaic cropland pixels (MCPs). NA, PA, HA and TA is short for no agreement, partial agreement, high agreement and total agreement respectively, which has the same meaning in the text.

The level of spatial agreement after the removal of MCPs can be clustered into three main types (Figure 6): (1) locally superior, i.e., concentrations of pixels with high agreement retained in typical irrigation areas, as mentioned in Section 3.1; (2) transition of pixels scored 2 to no agreement, which occurs in northeastern and northwestern China, caused predominantly by croplands emerging in Glob05 and GFSAD; and (3) total reduction in agreement, found in southeastern, southwestern, and some parts of the central region, which is induced not only by MCPs but also by fragmentation and dispersion of irrigation in GMIA and GRIPC.

## 4. Discussion

We found that GMIA shows the greatest coincidence with statistics, owing to numerical control during classification. The level of coincidence is reasonable but lower in GRIPC, although the map restricts the area amount. We suspect that this is caused by differences in the statistics used. MIRCA, to which GRIPC refers, is a database of main crop extent and AAI in 2000. Hence, we treated the quantitative difference between GMIA and GRIPC as demonstrative of the need for a unified database and classification system. True values, including field survey samples and visually interpreted images, have global coverage for irrigation mapping, especially in countries or regions with high dependence on irrigated agriculture. Nevertheless, values are collected disparately and for varying purposes. Moreover, most are collected and utilized only once at local scales. Information on crop plantations, such as cropping pattern and phenology, has been proven valid in massive irrigation mapping trials. Therefore, introduction of their datasets could greatly enrich the database because of their high extraction accuracies. Nowadays, there are platforms for field sample sharing with an operation pattern which is similar to crowdfunding, such as the Global Geo-Referenced Field Photo Library of University of Oklahoma (http://www.eomf.ou.edu.photos)—with collected land use, cover, and change information containing precise location and descriptions for real-time photos—but few attempts have been made on using the database in scientific researches.

However, Glob05 presents obvious discrepancies compared with GMIA, GRIPC, or neutral statistics, although it does possess a similar definition of irrigation to AAI, GRIPC, and GFSAD. A fractional assigning scheme with greater fit under quantity control might be the only way to reduce the level of disagreement. However, obtaining conversion ratios in locations with diverse conditions will be difficult. Since statistics have been taken into account in land use mapping, decomposition algorithms provide a possibility for improvement. The mathematic relation between remote sensed indices (such as NDVI) and the proportion of irrigated area in pixels have been discussed on the local scale [23,30,31]. However, the diversity of relationships also exists in different locations or indices [31], resulting in their localization. Thus, feature-selection and rule-definition have yet to be applied to the the field of irrigation mapping.

In general, a higher level of agreement implies higher spatial accuracy [32]. However, we were conservative in applying this within the context of our study, especially for locations with low levels of agreement. The coarse resolution used for the comparison might have caused omission of some spatial details in the converted maps. Meanwhile, it increased the impact of fragmentation in regions such as the southeast and southwest in terms of agreement scoring. Visual and spatial assessment of the levels of agreement of the maps with or without MCPs revealed that MCPs contain information that is both correct and incorrect. Unfortunately, identification of correct information is limited by the separability of the features or methods for mosaic classes. This demonstrates the need for clear and unified definitions of objective classes and for deeper comprehension of untypical places. Poor weather conditions, high levels of fragmentation, and multiple plantations could impede analysis. Improvement of classic methods or features might be difficult [33]. Even images with higher spatial or spectral resolutions, hierarchical strategy, and combination of supervised and unsupervised classification have been considered in some regional irrigation mapping studies [34,35,36]. Targeted exploration of new features and classification algorithms or tactics for irrigated cropland is still desired, for the ones referred in developing irrigation maps are inferior to those in similar cartography fields such as plantation and land cover [37].

## 5. Conclusions

Intercomparison of GMIA, GRIPC, Glob05, and GFSAD was conducted in quantitative and spatial phases in this study. We also investigated the contribution of MCPs in the latter two products. Quantitatively, GMIA and GRIPC exhibited similar numerical distributions in terms of the proportion of irrigated area. They also showed low dispersion and high goodness-of-fit with statistics, benefitting from numerical restriction during the classification. Notably, the four maps were spatially consistent in relation to typical irrigation areas, and the disagreement was contributed mostly by Glob05 and GFSAD. However, the accuracy of the maps in locations with low consistency might be affected by imprecise spatial information attributable to the coarse resolution used for the comparison. Visual comparison of maps with and without MCPs revealed their positive and negative effects.

The results of this study demonstrate the need for a unified database and classification system and for deeper discussion of untypical irrigated objects. The results also highlight the superiority of total quantity control in the classification. It is thus crucial to explore the following points: (1) the integration of existing data for a sharable database; (2) the use both of remotely sensed features and of algorithms for spatial reconstruction; and (3) the expansion of methods and features to deal with locations with multiple plantations, dimensions, and weather conditions.

## Figures and Tables

**Table 1:** Summary information of four datasets compared.

Dataset.	Benchmark	Resolution	Subclasses	Conversion Ratio
GMIA	2005	5 arcminute	1. Area of effective irrigation (AEI)	1
			2. Area of actual irrigation (AAI)	
GRIPC	2005	500 m	1. Rainfed croplands without irrigation or paddy	
			2. Croplands irrigated but without paddy	0.65
			3. Paddy with waterflooding for at least 2 weeks	0.65
Glob05	2005	300 m	1. Post-flooding or irrigated croplands	0.71
			2. Rainfed croplands	
			3. Cropland (50%–70%) with vegetation	0.6 × EP
GFSAD	2005	1 km	1. Cropland irrigated by any water resource	0.6
			2. Totally rainfed croplands	
			3. Mosaic croplands (40%–60%)	0.5 × EP

^1^ Classes assigned with conversion ratios are irrigation-relevant ones. ^2^ EP means provincial proportion of effective irrigation, quotient of statistical AEI, and cropland area. The conversion ratio of pure pixels is from Velpuri et al. [27]. The conversion ratio of mosaic pixels is the product of EP and average cropland ratio.

**Table 2:** RMSE, R, slopes and intercepts of regression analysis.

	GMIA	GRIPC	Glob05	GFSAD	Glob05-P	GFSAD-P
RMSE	0.50	1.37	3.05	1.97	4.47	1.34
R	0.96	0.80	0.29	0.46	0.32	0.75
Slope	0.95	1.06	0.76	0.70	1.18	0.91
Intercept	0.16	-0.19	0.78	0.96	-0.6	0.28

**Table 3:** Proportion of irrigated area accounted for by mosaic cropland pixels (MCPs) in Glob05 and GFSAD (%).

Datasets/Regions	Northeast	North	Southeast	Central	Southwest	Northwest	Total
Glob05	14.38	72.20	78.08	80.53	22.81	57.96	58.72
GFSAD	57.39	96.59	83.17	93.82	80.33	21.11	67.49
